# Aura and Head pain: relationship and gaps in the translational models

**DOI:** 10.1186/s10194-019-1042-8

**Published:** 2019-09-03

**Authors:** Hayrunnisa Bolay, Doga Vuralli, Peter J. Goadsby

**Affiliations:** 1 Department of Neurology and Algology, Gazi University Faculty of Medicine, Besevler, 06510 Ankara, Turkey; 2 Neuropsychiatry Center, Gazi University, Besevler, Ankara, Turkey; 3 Department of Algology, Bakirkoy Sadi Konuk Training and Research Hospital, Bakirkoy, Istanbul, Turkey; 4 Headache Group, Department of Basic and Clinical Neuroscience, King’s College London, London, UK; 5 NIHR-Wellcome Trust King’s Clinical Research Facility, King’s College Hospital, London, UK

**Keywords:** Cortical spreading depression, Migraine pathophysiology, Aura, Thalamus

## Abstract

Migraine is a complex brain disorder and initiating events for acute attacks still remain unclear. It seems difficult to explain the development of migraine headache with one mechanism and/or a single anatomical location. Cortical spreading depression (CSD) is recognized as the biological substrate of migraine aura and experimental animal studies have provided mechanisms that possibly link CSD to the activation of trigeminal neurons mediating lateralized head pain. However, some CSD features do not match the clinical features of migraine headache and there are gaps in translating CSD to migraine with aura. Clinical features of migraine headache and results from research are critically evaluated; and consistent and inconsistent findings are discussed according to the known basic features of canonical CSD: typical SD limited to the cerebral cortex as it was originally defined. Alternatively, arguments related to the emergence of SD in other brain structures in addition to the cerebral cortex or CSD initiated dysfunction in the thalamocortical network are proposed. Accordingly, including thalamus, particularly reticular nucleus and higher order thalamic nuclei, which functions as a hub connecting the visual, somatosensory, language and motor cortical areas and subjects to modulation by brain stem projections into the CSD theory, would greatly improve our current understanding of migraine.

## Introduction

Cortical spreading depression (CSD) is a pathophysiological phenomenon of gray matter characterized by sustained electrical silence and suppressed activity propagating in the cerebral cortex preceded by a brief shower of population spikes [1–3]. CSD has been shown to occur in traumatic brain injury, ischemic stroke and subarachnoid hemorrhage in the human cerebral cortex [4–8]. CSD has also been recognized as the biological substrate of migraine aura [9–11]. Experimental animal studies have provided mechanisms that possibly link CSD to the activation of trigeminal neurons mediating lateralized head pain [12]. The probable mechanism between CSD and lateralized head pain in anesthetized rats is over-extrapolated to migraine without aura and a ‘silent aura’ theory is proposed for migraine without aura to explain headaches. Some basic CSD features unfortunately do not match many clinical features of migraine headache well. Those disregarded grey areas are briefly discussed here in order to understand the exact role of CSD in migraine and to develop a new perspective into the mechanisms of the disease.

## What is the functional consequence of CSD at cellular level?

CSD is demarcated by a slowly propagating wave of depressed electrical activity in the affected cerebral cortical region [1, 2, 13]. The ‘depression’ or ‘silence’ is actually as a result of a state of sustained depolarization where the membrane is captured/clamped at the depolarized potential with loss of excitability for a few minutes. The electrical silence dominates in the cortical region as a prolonged membrane depolarization and prevents action potentials and synaptic transmission. Sudden collapse of ionic gradients across membranes is associated with massive movements of Na+, Cl-, Ca++ and water into the cell, K+ accumulation outside the cell, pH decrease to near ischemic levels and decrease of extracellular space by 50% [2, 13]. Such a toxic depolarization state of the tissue in CSD has nothing in common with depolarization of a neuron generating AP, except for the word of ‘depolarization’ itself (Table 1) [1–3, 13]. CSD happens in larger scale, lasts ~ 240,000 ms, causes up to 30 mV shift in the extracellular potential, covers every cell, and is characterized by loss of excitability of membranes in the tissue, while AP happens in micro scale, lasts 1 ms, causes no change in the extracellular potential, and is characterized by induced excitability state (Table 1) [1–3, 13]. The CSD process leads to an enormous release of intracellular contents, neurotransmitters, molecules, vasoactive and nociceptive substances including glutamate, GABA, aspartate, glycine, adenosine, catecholamines to the extracellular space in the depolarized cortical region. Such a massive movement of ions, water and substances across membranes change the extracellular potential and is reflected electrophysiologically as a huge transient shift up to 30 mV in direct current (DC shift) [1, 2]. Conventional alternating current coupled devices cannot detect these slow discharges unless the filter is changed to allow very low frequencies.

**Table 1:** Distinguishing features of depolarizations of action potentials and cortical spreading depression

	Depolarization of Action Potential	Depolarization of Cortical Spreading Depression
Duration	1 ms	Up to 345.000 ms
Membrane conductance	Increased passage of specific ions Na+, K+, Ca++ through their ionic gradients	Complete loss of membrane resistance and ionic gradients across cell membrane
Functional consequence	Excitation	Loss of function, no reactivity of the cell membrane, prolonged silence
Signal transmission	Axonal hillock to synaptic release of neurotransmitters to active postsynaptic neuron	Release of neurotransmitters and K+ and other molecules from cell and contiguous spread to neighbor cells
Extracellular DC potential change	None	Up to 30 mV
Recovery period	After a few ms	Reappearance of spontaneous APs takes minutes, spontaneous EPSP/IPSPs improvement lasts hours

The depolarization of CSD is reversible if the tissue is under physiological conditions and not associated with compromised energy/ blood flow. The contiguous spreading nature of DC shift comes from the fact that loss of membrane resistance and release of neurotransmitters, K+ and other molecules during depolarization induce a domino effect on adjacent cells. The improvement of electrical depression begins within a few minutes, though full recovery of spontaneous synaptic activity takes hours [2, 13].

The CSD induced depolarization state is not associated with increased excitability where action potentials fire, such as in epileptic discharges. On the contrary, the depolarization process of CSD is a loss of excitability state lasting minutes and very similar to the ischemic depolarization resulting in loss of function [2, 3, 13]. Whole-cell recordings in vivo confirm the prolonged depolarization up to 345 s with neuronal silencing during CSD, which was followed by reduced spontaneous synaptic activity and AP firing. Significant reduction in the spontaneous excitatory and inhibitory postsynaptic potentials, yielding an imbalance towards inhibitory tone, lasts longer than 90 min [13].

Consequently, the initial phase of CSD where the membrane resistance is lost and membrane potential is seized in depolarized state is a complete electrical silence that is unlikely to be associated with excitation of any cortical neuron leading to positive aura symptoms. On the other hand, a shower of population spikes lasting 2–4 s within the late gamma band that precedes DC drop [3, 14] could be attributed to positive symptomatology. However, the brief duration of antecedent spike shower may not be perceived consciously and may not be manifested as a clinical symptom. Even if it is perceived as a positive symptom, it has to be overwhelmed by following long-lasting negative symptomatology. Thereby, even the expectation of increased activity of excitatory cortical neurons with positive symptomatology alone is not compatible with basics of canonical CSD.

CSD was employed to model the depressed neuronal activity in the cerebral cortex and never been used to produce a brain hyperexcitability model. Transient loss of function of the cerebral cortex as a result of CSD was also noticed by other researchers and used as a method to generate functional decortication [15, 16]. The fact that CSD was used to produce functional decortication/ ablation [17, 18] indicates its massive negative functional consequences in mammalian brain. Recent studies unveiled the occurrence of SD waves in human cortex immediately before brain death, malignant ischemic stroke, aneurysmal subarachnoid hemorrhage or intracerebral hemorrhage and traumatic brain injury and their association with increased mortality and morbidity [4–8]. The latter conditions differ from migraine not only due to a severe disability but also the fact that majority of migraine auras consist of positive symptoms in visual, and/or somatosensory modalities.

## What is the evidence linking CSD to head pain?

The trigeminal nerve mediates the pain sensation from cephalic structures in the anterior portion of the head and plays a crucial role during headache phase of migraine. Peripheral trigeminal nerve endings arise from dural vasculature and therefore are termed as the trigeminovascular system. Upon activation, trigeminal nociceptors release their contents such as calcitonin gene-related peptide (CGRP) to perivascular space [11, 12]. Stimulation of trigeminal neurons at the ganglia, in experimental animals, results in neurogenic inflammation characterized by the release of CGRP and substance P, vasodilation, increase in blood flow and protein extravasation in the dura mater. Therefore, any activation of meningeal perivascular trigeminal nociceptors by potassium, arachidonic acid or other molecules released during CSD, could initiate signal transmission orthodromically to the pain processing structures, while inducing antidromic neurogenic inflammation in the dura mater in experimental animals [12].

In rat brain‚ CSD in pain-insensitive brain parenchyma was related to unilateral activation of trigeminal afferents and subsequent activation of 2nd order trigeminal pain neurons. The possible link between CSD and head pain was supported by the following findings in anesthetized rodent experiments; *1)* Two dimensional blood flow imaging of both the cerebral cortex and the dura mater revealed that following CSD wave, blood flow in the middle meningeal artery (MMA) notably increased for an hour in a trigeminal nerve dependent manner [12]. MMA was a perfect dural structure to observe the trigeminovascular system due to its dense innervation by trigeminal nerve. The increase in the ipsilateral MMA blood flow peaked 20 min after CSD and coincided with oligemic phase of the brain [12]. *2)* CSD triggered trigeminal nerve dependent plasma protein leakage, prominent around MMA within overlying dura mater in the ipsilateral hemisphere [12, 19]. *3)* CSD induced expression of the immediate early gene product c-FOS in trigeminal nucleus caudalis (TNC) in the ipsilateral brainstem [12, 19, 20]. Activation of the trigeminal nucleus also initiated trigemino-parasympathetic reflex which led to dural vasodilatation and hyperemia via release of vasoactive intestinal peptide, nitric oxide (NO) and acetylcholine, *4)* CSD could activate perivascular trigeminal nerve endings [21]. Those studies were conducted in rodents under anesthesia and suggested a link between an intrinsic brain event and lateralized head pain through peripheral bipolar neurons of trigeminal ganglia. Though the latter was disputed by Lambert et al. [22], where authors showed the persistence of CSD induced TNC neuronal activation following trigeminal nerve transection and thereby suggested a direct central pathway. Possible involvement of central route to activate TNC was also proposed to be operational particularly in awake subjects through thalamus and other brainstem structures [23]. CSD has also been shown to result in bilateral TNC activation [24, 25], decrease in mechanical pain thresholds in the head and pain-related behavior [25, 26].

CSD experiments in rodents showed the opening of pannexin 1 megachannels, MMP activation and BBB breakdown providing the possible mechanisms that explain how the intracellular nociceptive and vasoactive molecules induce neuroinflammation and find a way to pass through the blood-brain barrier and reach to the perivascular trigeminal nociceptors in the dura mater [19, 27, 28].

Another supporting evidence for the linkage of CSD to head pain came from the drug studies in rodents. Trigeminal activation at the second order neurons in TNC by CSD was reversed by sumatriptan [12, 20], valproic acid [23], topiramate [29], CGRP receptor antagonists [30] or metoclopramide [31]. Latter two agents also significantly reversed the CSD induced pain behavior such as grooming and freezing in addition to reduced von Frey thresholds and cold allodynia in freely moving rodents. It is important to note that acute use of aforementioned pharmaceutical agents drugs do not inhibit or influence CSD waves.

CSD is capable of inducing thalamic reticular nuclei (TRN) which is a key structure as a gate keeper in thalamocortical information flow and plays a role in selective attention, sensory discrimination, lateral inhibition, and sleep. TRN activation was in the visual sector and SD waves could propagate to TRN if the rats are devoid of any effect of anesthesia. The propagation of SD waves to TRN was blocked by valproic acid administration [23]. Therefore, its involvement in CSD is a very important step to look from a broader perspective into the cortical function since the cortical processing of any somatosensory, motor or cognitive function includes the thalamus, an inseparable counterpart of the cortex.

Transgenic mice expressing mutations associated with familial hemiplegic migraine (FHM) showed increased CSD susceptibility, where the glutamatergic synapse was the common target [32]. Reduced threshold for CSD induction and an increased CSD propagation speed were detected in both Cacna1aR192Q knock-in mice that carry the FHM1 mutation and FHM2 knock-in mice carrying the human W887R mutation in the ATP1a2 orthologous gene. Behavioral consequences of CSD in those mice exhibited several features attributed to migraine head pain and photophobia. However, their relation to trigeminovascular system or pain nucleus was not studied in mutant mice. Cacna1aR192Q knock-in mice displayed signs of photophobia and chose to remain in open space to avoid light in the bright closed arms condition and they spent significantly more time in the dark open arms than wild-type (WT) mice in modified elevated plus maze. Head grooming was increased in R192Q mutant mice and number of long strokes initiating in the oculotemporal region were significantly higher in Cacna1a transgenic mutants than WT animals showing a possible site of pain. Mutant mice also showed increased eye blinks associated with a whole-body shuddering behavior. Abnormal head grooming and eye behaviors were reversed by rizatriptan, a selective 5-HT1B/1D receptor agonist, an acute antimigraine medication in a dose-dependent manner [33]. In another study, Cacna1a knock-in mutant mice showed a baseline pain face with higher baseline mouse grimace scores compared to WT mice, which was prevented by intraperitoneal administration of 50 mg/kg rizatriptan [34].

## Which clinical manifestations suggest that CSD is the underlying mechanism of migraine with aura?

### The visual aura symptom matching the retinotopic organization of visual cortex is consistent with canonical CSD

Detailed imaging studies in nine migraine with aura patients showed that scintillations and other visual aura symptoms as well as their march are consistent with the retinotopy of the same individual [35–37].

### The gradual progress of aura symptoms is consistent with CSD

The gradual development of aura symptoms in approximately 30% of the patients, is also compatible with canonical CSD in humans. The gradual progress of visual aura was first related to occipital cortex by Lashley [10], who calculated the propagation rate of the underlying cortical process to be approximately 3 mm/min by assuming the length of striate cortex as 67 mm and the duration of enlarging scotoma to reach the margin of the temporal visual field from the center as 20 min.

Viana et al. [38] reported that, the somatosensory aura symptoms began after the end of the visual aura symptoms in 24% of the patients. This could be supportive for canonical CSD if the propagation time is long enough to reach somatosensory cortex (the details were not given in the study [38]. The presence of a time lag between the aura and the beginning of the headache which is seen in only 36% of the patients [38] is also compatible with the interval between CSD and subsequent development of trigeminal afferent activation.

### Contralateral manifestation of unilateral migraine headache and aura symptoms is in favor of canonical CSD in migraine

Regarding the bench studies, the CSD and headache is linked through the orthodromic activation of trigeminal nociceptors in the overlying dura mater and sequential induction of neurons in the ganglia and TNC [12, 19–22, 25]. According to the conventional notion, unilateral pain is expected to develop contralateral to the side of aura symptoms. In migraine patients the contralaterality is confirmed in a proportion of migraine with aura patients [39–44].

Manzoni reported that headache was contralateral to the somatosensory aura in 40% of his 164 patients [39]. In another study, 18% of 111 patients somatosensory aura was contralateral to the unilateral migraine headache [40]. Guiloff reported that only 1 patient out of 11, exhibiting contralateral distribution of paresthesia with headache [41]. Contralaterality of headache with somatosensory auras was around 35% in other series [42, 43]. Queiroz et al. reported contralaterality of migraine headache with visual aura in 8.2% of 122 patients [44]. Blood flow imaging studies in migraine with aura patients reported contralaterality in most of the subjects, though the number of patients included was limited. Therefore, the contralateral headache following aura symptoms is consistent with the canonical CSD features seen in rodents.

### Spreading hypoperfusion at a rate within established CSD limits is the main evidence supporting CSD in migraine aura

Vascular reactivity and blood flow alterations follow the change in neuronal excitability with a time lag and oligemia lasting for hours upon transient hyperemia is the typical feature of CSD. CSD usually induces hyperemia response in most of the species such as rats, cats or rabbits. Regional cerebral blood flow increase follows depolarization onset with a 16–20 s delay and peaks around 2 min after DC shift [2]. Then post CSD oligemia up to 40% starts and lasts hours in the affected cortical region.

While the direct electrophysiological evidence of DC shift or electrical silence has never been detected during aura in migraine patients, blood flow alterations are used to trace associated neurovascular events. Perfusion changes in migraine provide data supportive for a link between CSD and migraine aura. Hypoperfusion in the posterior part of the brain, spreading rostrally with a rate consistent with CSD propagation, is usually considered as an evidence for CSD in migraineurs’ brain.

During regional cerebral blood flow (rCBF) evaluation via intracarotid injections of radioactive xenon, occipital hypoperfusion advancing anteriorly was detected in images captured in 15 min intervals in 8 out of 250 patients (7 patients had migraine, 6 with aura, 1 without aura) [9]. When rCBF measurement was consistent with headache side, focal hyperemia was observed in 2 patients. Frontal hyperemia, followed by occipito-parietal oligemia was associated with contralateral paresthesia and ipsilateral fronto-temporal headache in one patient. Occipital hyperemia was replaced with global blood flow decrease in the other case. Lauritzen also reported reduced blood flow progressing anteriorly without crossing rolandic or sylvian sulcus with an approximately 1.8 to 2.6 mm/min rate in 7 patients [45]. Cerebral hypoperfusion affecting the whole hemisphere, sometimes with patchy involvement of occipital and frontal lobes was associated with the development of headache in migraine [45].

On the other hand occipital lobe hypoperfusion was reported during aura and before the onset of headache in 4 migraine patients with aura [46]. Similar oligemia changes were not detected in 13 migraine without aura patients [47], while bilateral occipital oligemia moving to temporal pole was demonstrated by Woods [48] in a well-known case of migraine without aura attack (Table 2).

**Table 2:** Comparison of unique aspects of cortical spreading depression with the findings in migraine

	CSD in rodents	CSD in primates	CSD in humans (TBI, SAH)	During Migraine Aura	During Migraine Headache Without Aura
DC shift accompanying electrical silence	+	+	+	Not detected ^a^	Not detected ^a^
ADC decrease DWI change	+	+	+	Negative even in FHM patients with neurological deficit lasting days	–
Propagation speed of DC shift	2–6 mm/min	4 mm/min	1.6–5.9 mm/min	–	–
Area occupied by DC shift	Whole cerebral hemisphere	Limited to a few gyruses	Limited to few centimeters	–	–
Spreading hyperemia	+ 20 s following a DC shift	+ Following a DC shift	+ Following a DC shift lasting 680 s	Focal hyperemic areas, focal oligemic areas and global oligemia (3 cases, *9*) Multifocal hyperemia (3 cases, 11 attacks, after 19 h during prolonged aura, *95*) Propagating increased BOLD response followed by reduced BOLD signal, (1 case, *35*)	–
Spreading oligemia	+ Few minutes Following a DC shift	No oligemia detected	+	Spreading oligemia towards the end of the visual aura (5 cases, *46*), Anteriorly progressing oligemia irrelevant to aura symptoms in time and location (*45*) Multifocal oligemia (3 cases, *95*) Spreading BOLD suppression (2 cases,*49*; 3 cases, *35*)	Spreading BOLD suppression (6 cases) (in case 3 right sided HA + suppression on the left side) (*49*) Bilateral spreading oligemia (1 case, *48*) Focal oligemia (2 cases, *92*)
Area occupied by hyperemia/oligemia	Whole cerebral hemisphere	Limited to a few gyruses	Limited to few centimeters	Global reduction in the hemisphere (*9*, *45*, *95*), occipital cortex (*35*, *49*)	Whole brain from occipital pole to temporal pole (*48*) Occipital lobe (*49*)
Propagation speed of hyperemia/oligemia	2–6 mm/min	NA	NA	2 mm/min, 3.1 mm/min 2.9–5.3 mm/min Detected during aura and end of aura (*35*, *45*, *49*)	3 mm/min

^a^ See text for comments on MEG DC shifts

Perfusion changes can occur due to neurovascular coupling mechanisms in response to neuronal depression. Suppressed BOLD activation was detected as initial change in the occipital cortex in migraine patients independent of the presence of aura and progressed at a rate of 3–6 mm/min to adjacent cortex in 8 patients [49]. Reduced BOLD signal was detected in response to visual stimulus during the scintillations and this reduction gradually progressed anteriorly from occipital lobe. BOLD suppression to visual stimulus spread at a rate of 3.5 mm/min and did not cross parieto-occipital sulcus. In addition, the scintillations were compatible with the retinotopy of the occipital cortex [35]. The latter case report provides the main imaging support for the existence of CSD during visual aura in humans. BOLD amplitude reduction was also detected during a migraine visual aura consisting of a scotoma with flickering zig zag border and was considered as a supportive finding for CSD [37].

In conclusion, variable degrees of oligemia are reported during migraine aura symptoms in a distribution correlated with clinical symptoms. However, significant data also shows that oligemia and aura symptoms occur independently, as focal, global/bilateral hypoperfusion may accompany monosymptomatic auras or can be detected without any clinical symptoms of aura. Moreover, migraine headache without aura is reported to be associated with spreading occipital oligemia [48, 49] or with no perfusion defects at all [47].

### Drug effects on CSD induced TNC activation and CSD susceptibility provides further supportive evidence for CSD in migraine

Acute administration of migraine abortive drugs, such as sumatriptan or CGRP receptor antagonists, reduce CSD induced TNC activation in rodents [12, 20, 30]. None of the available drugs can completely block the CSD waves, however, they reduce the number and amplitude of CSDs and alter the CSD threshold. Abortive medications such as sumatriptan do not have any effect on CSD [50] but able to reduce the c-fos activation in TNC induced by CSD.

Preventive drugs are shown to reduce CSD susceptibility in vivo even though they have different mechanism of actions. In a study that evaluated the effectiveness of anti-migraine agents in the prevention of CSD using laser Doppler flowmetry in cats, a single dose of dihydroergotamine, acetylsalicylic acid, lignocaine, metoprolol, clonazepam or valproate was not able to inhibit CSD, did not reduce the propagation rate or alter the amplitude of the increase in cortical blood flow [51]. However, in another study, a single dose of topiramate was shown to prevent mechanically-induced CSD 30 min after the administration [52]. On the other hand, chronic administration of valproate, propranolol, amitriptyline, topiramate and methysergide dose dependently reduced CSD frequency and increased the electrical stimulation threshold [53]. Furthermore a later study also confirmed that chronic administration of topiramate (once-daily for 6 weeks) inhibited KCl-induced CSD frequency and propagation [54].

Lamotrigine suppressed CSDs in rat brain [55], and reduced migraine with aura attacks in open-label un-controlled studies however, it did not exert any preventive effect in migraine without aura patients in controlled trials [56–60]. Tonabersat, an antiepileptic drug with prominent CSD and gap junction inhibiting properties was shown to have preventive effect on attacks of migraine aura [61] however it had no clear benefit on migraine headache days [61, 62]. A NMDA receptor blocker MK-801 was shown to inhibit CSDs in rodents. Another NMDA receptor antagonist, ketamine, was found effective in the treatment of prolonged aura symptoms but not the headache [63, 64].

## What are the gaps in translating CSD to migraine with aura?

CSD could explain some features of migraine but following unresolved points needs to be addressed.

### Occurrence of migraine headache ipsilateral to the visual and /or sensorimotor aura symptoms cannot be explained by CSD in the human cerebral cortex

In rat brain CSD in pain insensitive brain parenchyma was linked to unilateral headache development through the activation of dural perivascular trigeminal nociceptors overlying the cerebral cortex, and neurons in the trigeminal ganglion and trigeminal nucleus caudalis all in the same hemisphere [12]. Trigemino-autonomic reflex arc was also induced by CSD in the same hemisphere. The study provided a mechanism to understand how aura and headache are related through peripheral pathway by trigeminal nerve mediated blood flow increase in MMA and dural neurogenic edema in anesthesized rats. By applying this theory to human conditions, aura symptoms as a result of CSD in one hemisphere are expected to occur contralateral to the headache side. In clinical practice this is not the rule and significant proportion of patients exhibit symptoms that do not meet that assumption. In fact, aura symptoms originating from one hemisphere and unilateral migraine headache located to the other hemisphere were reported in many clinical studies. Studies reporting ipsilateral distribution of neurological dysfunction and headache are summarized below.

- In a detailed study of 111 migraine with aura patients, Peatfield and colleagues reported that somatosensory aura symptoms such as paresthesia were on the same side as headache in approximately half of the patients [40]. Fifty percent ipsilaterality is a very high proportion particularly for somatosensory aura symptoms as its laterality is more reliably defined. When all aura symptoms were considered, about two-thirds of 111 patients manifested neurological symptoms originating from one cerebral hemisphere and headache developed in the opposite hemisphere [40].
  Manzoni and colleagues published a study in which paresthesia was reported by 30% of 164 patients and most of them had a cheiro-oral distribution [39]. Unilateral paresthesia was ipsilateral to the headache in approximately 40% of 49 migraine patients with sensory aura [39]. Similar ipsilaterality of somatosensory symptoms and migraine headache was also notable in 4 out of 9 cases reported by Lauritzen [45], where rCBF was measured by Xenon through an intracarotid catheter. Paresthesia and numbness ipsilateral to the headache side were reported in 23% of 84 patients with somatosensory aura in the study conducted by Bruyn et al. [43] and in also other series [41, 42]. Unfortunately, the laterality of 162 aura symptoms and headache was not given in a prospective study [38].
  Among 76 unilateral migraine headache and visual auras, headache was ipsilateral to the visual aura symptoms in 22% [44]. Though visual aura and migraine headache contralaterality was reported in only 8–16% of the patients [44, 65].

b) Language is lateralized to the left hemisphere in most right handed individuals and aphasia due to right hemispheric disorder in a dextral person is called crossed aphasia which is a rare condition with a prevalence of 1–4% in healthy individuals. Martins reported 7 right handed migraine with aura patients who developed left sided paresthesia and/or hemianopsia in addition to aphasia [66]. Nine out of 22 right handed dysphasic migraineurs had both headache and paresthesia on the right side, 5 exhibited both symptoms on the left side. Only 2 right handed dysphasic patients developed left sided migraine headache and right sided paresthesia [40]. Language problem of aphasic type occurred as an aura in 28 migraine patients and aphasic disturbances were contralateral to the prevailing hemisphere in 30% of patients studied by Manzoni and colleagues [39].
  Sensory aura symptoms on the left side accompanied by dysphasic symptoms were also defined by others [45]. These findings are not compatible with the notion that aura symptoms and headache are due to canonical CSD in one cerebral cortex.
  Above reports indicate that the theory of ‘typical CSD in human cerebral cortex cause unilateral headache via direct activation of overlying dural trigeminal nerve endings and trigeminal neurons in the same hemisphere’ has flaws to explain migraine with aura attacks in significant proportion of patients. The ipsilateral localization of headache and aura symptoms do not match canonical CSD features.

### Bilateral aura symptoms cannot be justified by canonical CSD in the human cerebral cortex

Propagation of CSD requires intact contiguous neural elements in the gray matter and is limited by subcortical white matter in addition to major sulci and large arteries in gyrencephalic brain. Once initiated, CSD propagates within the same hemisphere [12, 13, 67]. In Leao and Morison’s [68] experiments in rabbit brain‚ stronger electrical stimulation in the frontal cortex was able to induce depressing effect in the homolog area via crossing axons. This phenomenon is dissimilar to the propagation of conventional CSD to contralateral hemisphere and authors concluded that it was immediate result of the electrical stimulus passing through the crossing fibers in the callosum. Leao [1] reported that ‘a wave of depression started in the stimulated hemisphere and did not spread to the opposite side’. There is no report for canonical CSD propagating to the other hemisphere. On the contrary, many studies concluded that CSD did not affect the other hemisphere [1, 8, 12, 13, 19, 20, 23, 30, 67].

Therefore, the manifestation of bilateral aura symptoms cannot be explained by CSD crossing the callosal fibers to the other hemisphere. Canonical SD in the cortex does not invade two separate cortical sensory areas simultaneously.

### Emergence of visual and somatosensory auras simultaneously or sensory auras that begin in the visual field and continue with somatosensory and language dysfunctions cannot be justified by the propagation of canonical CSD in the human cerebral cortex

In great majority of patients presenting with both visual and somatosensory aura, visual disturbances appeared first and paresthesias appeared later. Recent records of 162 auras prospectively collected [38] showed that single aura symptom was visual in 97% of the patients, while auras consisting of two symptoms were visual followed by somatosensory in 81% of the patients. The second symptom started concurrently with the first aura symptom in 35% or on the course of the first symptom in 37% of the patients. Only in 5% of the patients, did the second aura started after the first symptom ceased [38]. Regarding the duration, most of the visual aura symptoms (≈60%) lasted between 11 and 30 min [38]. Manzoni and colleagues reported that the average duration of aura was less than 30 min in 76% of the cases [39]. Dysphasia symptom, always followed visual and/or sensory auras, generally lasted less than 10 min [38]. For instance, hemianopsia and aphasic aura characterized by anomia and semantic paraphasia lasted 10 min in migraine patients [59].

Furthermore, considering the distance that CSD has to travel with the time interval between aura symptoms the issue gets more complex. Even in a flattened macaque brain the distance from V1 to somatosensory cortex is 100–120 mm [69]. The distance between occipital pole and central sulcus in surface reconstructed/flattened human cortex is approximately 250 mm [70]. A wave of CSD from visual areas to sensorimotor cortex or language areas should march faster than 10–25 mm/min. Such a high speed was never detected in any species. The usual propagation rate is between 2 and 6 mm/min for any CSD in mammalian cerebral cortex, which would take approximately 42 to 125 min for CSD to travel such a distance. The concomitant manifestation of visual and somatosensory aura symptoms is inconsistent with march, although simultaneous manifestations of CSD at two non-contiguous sites remains possible.

Additionally, the somatosensory symptoms typically manifest as cheiro-oral syndrome (ipsilateral fingertips and per oral distribution). Cheiro-oral somatosensory aura symptoms start as tingling sensation or pins and needles from acral part of digits and propagate to wrist, and involve lips and tongue. In the cortical sensory homonculus, each digit is oriented in a caudo-rostral direction with 10–20 mm apart, where the acral part lies rostrally and there is 20–30 mm distance between face area and digits [71, 72]. Prospective studies also showed that somatosensory aura follows and/or accompanies visual aura in great majority of attacks [38]. Accordingly, the involvement of distal parts of the fingers and perioral area, tongue and palate while skipping the head, forehead, eyes, and nose in the cortical sensory homonculus seems impossible, as CSD waves are considered to travel rostrally from occipital cortex in a continuous manner. This is not compatible with hand and finger somatotopy in the cortical homonculus [72].

Canonical CSD propagating through the cortex would also be expected to invade other areas between visual and somatosensory cortex and yield symptoms such as acalculia, agraphia, finger agnosia, and right–left disorientation, contraversive eye deviation, buzzing noise in regard to data from cortical stimulation studies in human [73]. The majority of somatosensory aura last 11–20 min [38]. In the light of the above data, somatosensory symptoms starting simultaneously with visual aura symptoms or while visual aura symptoms still continue, do not match the known properties of canonical CSD phenomenon, unless they start in more than one place at the same time, triggered by other events. For example, decreased cortical noradrenergic signaling from locus ceruleus (LC) was shown to reduce the threshold for CSD induction [74]. Considering the pontine dysfunction in the premonitory phase and noradrerenergic projections to the entire brain, LC involvement could provide explanation for some clinical features.

If a SD like event travels in a restricted manner along a gyrus or sulcus, how does a CSD in the visual cortex manifest as a visual aura and travel to the somatosensory cortex causing paresthesia within 20 min? Or how do visual and somatosensory cortical dysfunction occur simultaneously?

CSD is limited to a few gyri in the primate brain however for multiple aura symptoms to occur sequentially, CSD should propagate over numerous sulci along the human cerebral cortex. Simultaneous occurrence of visual and somatosensory auras in the majority of patients needs to be explained with a process other than spreading depolarization waves continuously traversing along the cerebral cortex.

### Canonical CSD in human cerebral cortex during migraine aura has not been proved electrophysiologically

DC shift, suppressed ECoG/ electrical silence and consequent loss of neurological function, lasting minutes, within the involved cerebral cortical area are key features of CSD. CSD is hardly induced in healthy, metabolically uncompromised primate brain [75–77]. This is opposite to the experimental findings in lissencephalic brains where CSD can easily be induced by many ways and invades the whole hemisphere. Many patients with awake cortical mapping reported no dysfunction lasting minutes resembling CSD. CSD or its functional consequences were not reported in those awake neurosurgical procedures [76]. However even electrophysiological mapping studies of the human cortex in epileptic /tumor surgery did not reveal any phenomenon resembling canonical CSD seen in rodent brain [78]. McLachlan reported that he could not elicit CSD in the human cerebral cortex of 23 patients using chemical, mechanical or electrical stimulation including intracortical KCl injection while similar approaches easily induced CSD waves in the rat brain [75]. In humans, SD waves on the other hand are easily recognized in damaged cerebral cortex due to hemorrhage, trauma or severe ischemia [6]. Even if CSD is induced, its propagation is easily blocked or limited to a few gyri in primates and humans [36, 77]. Indeed, mapping of a migraine aura onto the visual cortex suggests that the event underlying visual aura can travel along a single gyrus or sulcus [36]. Likewise, electrophysiological hallmarks of canonical CSD have not been detected convincingly in migraine.

MEG DC shifts were reported in migraine brain while they were also detected in the control group with lower amplitudes [79, 80]. However it was barely convincing that those changes were due to an underlying CSD in the occipital cortex. MEG DC shifts were not specified to the aura period but were also observed during headache and interictal periods [79]. The aura symptoms and their duration, lateralization and retinotopic correlation were not defined in the subjects and spreading nature were not shown [79, 80].

MEG DC shifts were widely distributed in both hemispheres particularly in occipito- parieto-temporal cortices in migraine patients. During the spontaneous attacks in migraine with aura patients, greater activation was detected in the occipito-parieto-temporal association cortices instead of primary visual cortex, though, the visual stimulation induced activation was clearly seen in the occipital cortex [80]. MEG DC shifts were more pronounced in the occipito-parietal-temporal, higher order association cortices while occipital cortex has very low activation even during the early stages of aura/HA [80]. Those changes therefore cannot indicate an underlying CSD.

Detecting MEG DC shifts alone cannot be taken as a proof of CSD, considering the fact that DC shifts in the MEG and /or EEG are also detected during cortical activation by auditory stimuli [81], cognitive tasks, changes in vigilance states, changes in brain CO2 levels, shifts of attention, hyperventilation [82], hemodynamic changes [83, 84] sensory stimulation, finger movements [85] or epileptic seizures [86–88]. MEG DC shifts have a good localizing value for epileptic seizures and they are timely locked to neuronal activity in the higher frequencies. Infraslow oscillations in the DC range are believed to represent a slow modulation of gross cortical excitability [89]. Additionally generation of infraslow oscillations likely involves thalamic networks and/or astrocytic calcium waves [90, 91]. MEG DC shifts in the migraine brain could indicate cortical hyper-excitability and also be associated with these factors.

The failure to show electrical imprint of CSD in migraine patients could be related to the fact that CSD is limited to a relatively few number of gyri in human cortex under normal physiological conditions. The latter is somehow contradictory, when oligemia progressing almost in the whole hemisphere is accepted as a result of CSD in migraine brain.

### The occurrence of aura and headache without any temporal relationship, or aura without any pain, is not compatible with conventional notion of CSD

If the CSD induced depolarization occurs only a single or a few gyri in human brain, the area of activated trigeminal perivascular nerve endings in the overlying dura mater would be inadequate to fire trigeminal neurons for the development of lateralized head pain.

In animal models nociceptive and vasoactive substances released during CSD diffuse to the dura mater and induce trigeminal nerve endings and lead to the activation of the trigeminal ganglion and trigeminal 2nd order pain neurons in the ipsilateral hemisphere. However, most of the migraine attacks occur without aura and aura may occur without headache in 10% of patients [38, 44]. Furthermore, headache may even start before aura (9%) or emerge simultaneously with aura (14%) [38]. A prospective study reported that in only 36% of the attacks, headache started with an interval after the end of aura [38]. Prospective clinical studies also prove that aura and headache can occur any time and no temporal relationship exists between them [36, 38]. CSD in the gyrencephalic cerebral cortex is limited to a few gyri and can only activate a very limited portion of trigeminal nociceptors in the dura mater and MMA, therefore headache development secondary to the aforementioned process is very unlikely.

### CSD associated vascular reactivity changes do not match the rCBF/BOLD alterations detected in migraine patients

It is critical to note that CSD induced vascular reactivity has species differences. For example, initial rCBF decrease accompanies DC shift in mice, followed by hyperemia phase and post SD oligemia. On the other hand, focal hyperemia was recorded in primate CSD models while long-term hypoperfusion/or persistent hypoperfusion was not detected [77]. Additionally, in vivo whole-cell recordings showed that the neuronal silencing after CSD was due to reduced spontaneous synaptic activity and AP firing. Significant reduction in the spontaneous excitatory and inhibitory postsynaptic potentials lasted more than 90 min [13] yielding an imbalance towards inhibitory tone. Therefore, the hyperemia phase coincides with the neuronal silence in the cerebral cortex. In other words, if the hyperemia detected in the migraine brain is due to a canonical CSD, then it should be associated with suppressed neuronal activity and possibly negative symptoms. Moreover, if the initial depolarization phase is missed by EEG studies then the detected hyperemia phase should be associated with negative symptoms.

Lauritzen and colleagues reported clinical features and rCBF measurements in patients who developed migraine with aura attacks after the introduction of a catheter into the internal carotid artery. Spreading occipital oligemia observed in all patients with approximately at a rate of 2 mm/min did not cross the lateral or central sulcus but appeared in the frontal lobe as well. Remarkably hemispheric blood flow changes were not correlated with lateralization, localization and /or beginning of aura symptom,e.g. during the oligemia spreading through the temporal, parietal and frontal lobes, paresthesia was the only symptom, oligemia lasted quite longer than the focal neurological symptoms or the left hemispheric rCBF changes resulted in left sided focal symptoms [45]. Therefore, spreading oligemia was concluded not to be associated with aura symptoms spatially and temporally and not to be the cause of focal symptoms in migraine aura [45]. So, oligemia begins before the aura and outlasts the aura symptoms and has no resemblence with CSD except for the propagating speed of approximately 2 mm/min [9, 45].

Later studies reported reduced cerebral blood flow, volume or perfusion in the symptomatic occipital cortex during visual aura [46]. Blood flow changes were usually not detected in migraine patients without aura [47], although Gelmers et al. reported focal oligemia in two migraine without aura patients [92] and Woods et al. reported bilateral occipitotemporal oligemia in one migraine without aura patient [48].

Apparent diffusion coefficient (ADC) decrease with DC shift reflects water movement during cellular depolarization. Transient regional hyperperfusion and post-CSD oligemia were also demonstrated by MRI in rats [93]. ADC decline and diffusion-weighted imaging (DWI) changes reported during CSD in animal studies [93, 94] were never detected in human studies even when rCBF was reduced to 53% during a visual aura or in FHM patients with prolonged neurological deficits [46, 95]. Similarly, BBB remains intact during migraine attacks in contrast to the CSD experiments in rodents [28, 96].

The majority of migraine patients exhibit positive visual and somatosensory symptoms [38, 65, 97, 98]. Positive visual symptoms were reported in 89% of the migraine patients [65]. Even though positive symptoms constitute the majority of aura symptoms, negative aura symptoms may also occur. Viana et al. reported [38] that 67% of visual aura included at least one positive phenomena whereas 38% of visual aura included at least one negative phenomena and 14% of the patients had negative sensory symptoms. In another study [99] blind spots were observed in 42% of the patients and tunnel vision was seen in 27%. Hartl et al. reported positive visual symptoms in 55.6% of the migraine patients while negative visual hallucinations were reported in 51.9% [97]. However, CSD research in lissencephalic brain show that light induced neuronal activity or synaptic evoked activity is reduced in parallel to the blood flow decrease manifesting negative symptoms. Actually, during the positive symptoms experienced as scintillations, BOLD response in the visual cortex is reduced [34, 49]. BOLD signal, an indirect measure of neuronal activation, reflects the excitatory and inhibitory post synaptic potential activity rather than spike activity. Increased synaptic activity leads to a positive BOLD response while decrease in local neural activity results in negative BOLD signal in the cerebral cortex [100–102]. Neural activity decrease is usually related to the activity of inhibitory interneurons [103] which is also compatible with the findings of whole cell recordings during CSD [13].

BOLD response to visual stimulus was decreased bilaterally, pronounced in V1, in a patient who developed flickering visual symptoms on the right side and throbbing feeling in the right hand followed by pricking sensation in the right arm. This is another example of dissociated aura symptoms and BOLD response in migraine [37]. Remarkably BOLD responses were increased in both hemispheres after sumatriptan, a drug that has no effect on CSD [37].

Both oligemia and hyperemia were shown in FHM2 patients. Multifocal hypoperfusion was prominent within the first 19 h (in 71.4% of the attacks) when the aura symptoms and headache were both present, and hyperperfusion developed after 18 h when neurological deficit still persisted and the headache was resolved in 89% of the attacks. Multifocal hypoperfusion detected during acute phase of hemiplegic migraine with prolonged aura is not compatible with canonical CSD propagation in the human cerebral cortex [88]. Cerebral metabolic rate of glucose (CMRGlu) increases with the DC shift in CSD experiments and returns to normal levels after about 15 min [104]. In FHM2 attacks, increased cerebral glucose metabolism was detected by FDG-PET in the regions corresponding to the areas of hyperperfusion on day 4 while aura symptoms still persisted.

Hypoperfusion as detected by delayed rMTT and TTP and decreased rCBF were reported in 14/20 migraine with aura patients who admitted to ER with the diagnosis of stroke [105]. Perfusion defects usually exceeding a single vascular territory, bilateral hypoperfusion (3 out of 14 patients) or whole hemispheric hypoperfusion resembling to severe stenosis of extracranial vessels (4 out of 14 patients) were detected in migraine patients. In a child case of confusional migraine attack with conjugate eye deviation, ASL CBF image revealed a reduction in rCBF in right occipito-parietal blood flow to 34 mL/min/100 g while rCBF was 210 mL/min/100 g on the contralateral occipito-parietal region [106]. ASL image of a migraine attack with bilateral headache revealed hypoperfusion in bilateral thalamus and hypothalamus and hyperemia in bilateral frontal convexity [107]. On the other hand MR imaging of 4 migraine patients during aura attacks did not reveal any abnormality in cerebral perfusion [108]. In Woods’ case, during a migraine without aura attack, bilateral hypoperfusion started from occipital pole gradually invading more rostral areas to temporal cortex [37, 48]. The PET images taken during a visual stimuli for 3 h showed an estimated blood flow decrease up to 40% which was not associated with any positive or negative neurological deficit or caused any aura symptoms on the way from occipital V1 to temporal pole [48]. Remarkably, oligemia propagated easily through parietooccipital sulcus and other sulci without any problem.

Vascular reactivity changes as an indirect way to observe neuronal changes does not provide a rational explanation for the occurrence of CSD or as a cause of CSD. Hemispheric oligemia solely cannot be accountable for a migraine headache development. The perfusion changes during and following migraine attacks seem to be variable though the predominant feature in the aura phase is accepted as a hypoperfusion which may extend into the headache phase. Hyperperfusion is proposed to occur during headache (HA) phase, which is also not a consistent finding.

Original rCBF studies did not support the notion that oligemia was associated with aura, on the contrary, it was concluded that oligemia propagating from posterior to anterior accompanied headache [9, 45, 48]. It seems that reduced blood flow alterations are not primarily responsible for aura symptoms. Neither hypoperfusion nor hyperperfusion are thoroughly correlated temporally or spatially with aura symptoms or headache. So the rCBF studies do not support a canonical CSD as an underlying cause and remain inconclusive for the development of migraine headache. The above arguments indicate a need for a more complex theory to rationalize those changes.

### If the CSD induced activation of trigeminal nerve fibers around the MMA plays a key role in head pain development, then the migraine headache is expected to be localized more in the mandibular or maxillary branch of trigeminal nerve in humans

MMA particularly the proximal parts is densely and exclusively innervated by mandibular and maxillary branches of trigeminal nerve in humans [109–111]. The receptive field of the first branch, ophthalmic nerve does not include the proximal part of MMA. However, migraine headache is characterized by localization of pain to ophthalmic branch of trigeminal nerve and upper cervical nerves. Though some studies actually reported migraine pain in lower face [112], the MMA may not be a main determinant in migraine as it is shown in rodents. Supporting the latter notion, in spontaneous or provoked migraine attacks no dilation of extracranial arteries or MMA were detected in migraine patients [113, 114]. In cilostazol induced migraine attacks [115] increase in MMA was considered as a possible marker for activation of dural perivascular nociceptors, suggesting a meningeal site of migraine headache. Since the typical migraine headache is localized to the ophthalmic branch, C1 (intracranial) and C2 distribution (posterior part of the head), what would be the meaning of the MMA diameter change? If the MMA and/or its trigeminal innervation is a determinant in migraine then why the headache is not usually perceived in the maxillary or mandibular branch? CSD initiated sequence of events through dural trigeminal nerve fibers seems to have no translational correlate in humans.

### Aura symptoms are not specific to migraine headache

Typical aura symptoms can accompany other conditions and primary headaches. For example presence of aura symptoms is reported in up to 25% of cluster headache patients [116, 117]. Their existence in other primary headaches lessens its causal relationship with migraine headache and suggests a possibility that aura could be an epiphenomenon in primary headaches.

### CSD suppression is not required for effective migraine prevention

Recent progress on CGRP receptor antagonism by monoclonal antibodies that are effective in the prevention of both episodic and chronic migraine is very important. The monoclonal antibodies against CGRP or the CGRP receptors pass the blood-brain barrier minimally and are believed to exert their effect on the peripheral trigeminal structures. Up to 25% of patients are reported being 100% headache free for months. Those findings indicate that central mechanisms may not be required or CSD is not a triggering factor at least for those subgroup of patients. The tonabersat trial does not favor an initial role of CSD in migraine headache development in humans. Failure of patent foramen ovale (PFO) closure in migraine headache prevention is also notable since PFO is regarded as a triggering factor of aura [118].

Though many efficient migraine preventive agents are shown to reduce the frequency of KCl induced multiple CSD occurrence, CSD reducing properties are not predictive for drug effectivity in migraine prevention.

## Alternative view to explain translational gaps of CSD in migraine

Hence the concept of canonical CSD waves that propagate strictly within the human cerebral cortex and cause aura symptoms and headache, has several shortcomings to justify manifestations of the migraine disorder, additional mechanisms and views are needed.

CSD could still occur in a restricted area in the migraineur’s cortex where the propagation of depolarizating waves are easily stopped and terminated, as shown in human cortex. However, the consequence of an unresponsive cortical focus should be taken into account in a context with thalamocortical oscillations and widespread brain network systems. Understanding of cerebral cortical function requires a knowledge of complex interaction of the thalamocortical network. The thalamus sends projections to almost all cerebral cortex, and the cortical region receiving projections from the thalamus, sends outputs to one or more thalamic nuclei, in return [119–121]. In that sense the thalamic nuclei can be considered as a first-order and higher-order relay nuclei. First-order thalamic nuclei, such as the lateral geniculate nucleus (LGN) and the ventral posterior (VP) nucleus, receive and relay inputs from ascending visual and somatosensory pathways to first order cortical areas [119, 120]. On the other hand, higher-order thalamic nucleus, receive inputs principally from the 5th layer of the cerebral cortex and relay information between different cortical areas [120]. Thereby, transthalamic cortico–cortical circuits can synchronize oscillations over cortical distances [18, 121].

Thalamus and cortex are in a continuous interaction in processing any information particularly related to sensory, motor, language, cognition and consciousness. Abrupt silence of cortical counterpart as a result of SD would result in an imbalance that needs to be filled/adjusted with the change of usual firing pattern of thalamic nuclei. CSD is estimated to be induced if a stimulus simultaneously depolarizes a minimum critical volume of 1 mm^3^ in rodent cortical brain tissue [122]. Even if its propagation is blocked, such a cortical focus is sufficient to create a change in ongoing cortico-thalamic drive on thalamic relay nuclei and thalamic reticular nucleus. For instance, sudden loss of excitatory drive on GABAergic TRN that inhibits other thalamic nuclei, would result in increased sensory excitation, reduced discrimination and reduced lateral inhibition. The latter findings were shown in migraine patients [123, 124]. In episodic migraine patients, a transient but robust prolongation in somatosensory temporal discrimination thresholds (STDTs) was shown during the migraine attacks whereas STDTs were normal in tension-type headache patients [123, 125]. The discrimination of the exact entry of sensory stimuli was disrupted in migraine attack, indicating an impairment in central somatosensory and cognitive processing [126].

The pulvinar in the dorsal thalamus contribute to the visual information processing and multimodal sensory integration [102]. A dysfunction in higher order thalamic relay nucleus such as pulvinar dysfunction may be related to positive visual hallucinations superimposed to a primary incoming information and could be more compatible with the patients’ visual auras. High levels of connectivity of pulvinar with the primary motor-sensory cortices, superior parietal gyrus, precuneus and visual cortex [127] are in favor of the latter view. Recently impaired sensorimotor integrity and reduced cortico-cortical inhibition between somatosensory and motor cortices were shown to be associated with migraine attacks [128]. Notably, facilitation of motor responses to a conditioned sensory stimulus was detected even several hours prior to headache onset. Therefore, the contribution of higher order thalamic nuclei to these changes seems irrefutable.

It is also probable that SD ignition in the cortex could induce SD in TRN [23], as subcortical structures are still relatively more vulnerable to SD compared to highly convoluted cerebral cortex in humans [129]. Majority of somatosensory aura symptoms are cheiro-oral type (paresthesia in the fingertips and perioral area, tongue and palate). Majority of cheiro-oral syndromes has been associated with thalamic or brain stem lesions [130]. Likewise, in the thalamic ventral posterior (VP) nucleus, the representation of the acral parts of all digits, the tongue and the lips in close proximity, is more compatible with paresthesia starting from distal parts of all digits moving proximally skipping upper arm, shoulder, head, forehead, eyes and nose [131]. Additionally, aphasia can also occur as a result of thalamic dysfunction [132] and thalamus was implicated in migraine [23, 133, 134]. Pontine and other brain stem projections from cholinergic and noradrenergic nuclei, LC to the thalamus provide multiple modulatory drive on thalamo-cortical interactions. Thalamic player in accordance with the cortex would enlighten positive aura symptoms and concurrent manifestation of different symptoms related to certain cortical areas such as visual, somatosensory, language and motor cortices connected through thalamic hub.

## Conclusion

Migraine headache is a heterogeneous complex disorder and it seems implausible to propose single mechanism to elucidate sufficiently all clinical manifestations. Therefore, more than one mechanism and multiple locations must be considered in the development of migraine headache. While the canonical CSD could explain visual aura and following headache in certain population of patients, it has flaws in reasoning multiple sensory auras. It is also important to account thalamus as a hub that provides extensive connections among the visual, somatosensory, language and motor cortical areas. Emergence of SD in reticular nucleus or other locations in the thalamus could thereby rationalize some features of migraine. Additionally, including central pathways activated by thalamocortical dysfunction in the process of headache development would improve our understanding of migraine.

## Data Availability

Not applicable.

## Abbreviations

ADC: Apparent diffusion coefficient
CGRP: Calcitonin-gene related peptide
CSD: Cortical spreading depression
DC: Direct current
FHM: Familial hemiplegic migraine
HA: Headache
LC: Locus ceruleus
MMA: Middle meningeal artery
NO: Nitric oxide
rCBF: Regional cerebral blood flow
STDT: Somatosensory Temporal Discrimination Threshold
TNC: Trigeminal nucleus caudalis
TRN: Thalamic reticular nucleus
WT: Wild type
